# The impact of mouth breathing on dentofacial development: A concise review

**DOI:** 10.3389/fpubh.2022.929165

**Published:** 2022-09-08

**Authors:** Lizhuo Lin, Tingting Zhao, Danchen Qin, Fang Hua, Hong He

**Affiliations:** ^1^Hubei-MOST KLOS & KLOBM, School & Hospital of Stomatology, Wuhan University, Wuhan, China; ^2^Department of Orthodontics, School & Hospital of Stomatology, Wuhan University, Wuhan, China; ^3^Center for Dentofacial Development and Sleep Medicine, School & Hospital of Stomatology, Wuhan University, Wuhan, China; ^4^Center for Evidence-Based Stomatology, School & Hospital of Stomatology, Wuhan University, Wuhan, China; ^5^Division of Dentistry, School of Medical Sciences, Faculty of Biology, Medicine and Health, The University of Manchester, Manchester, United Kingdom

**Keywords:** mouth breathing, malocclusion, maxillofacial development, adenoids, palatine tonsil

## Abstract

Mouth breathing is one of the most common deleterious oral habits in children. It often results from upper airway obstruction, making the air enter completely or partially through oral cavity. In addition to nasal obstruction caused by various kinds of nasal diseases, the pathological hypertrophy of adenoids and/or tonsils is often the main etiologic factor of mouth breathing in children. Uncorrected mouth breathing can result in abnormal dental and maxillofacial development and affect the health of dentofacial system. Mouth breathers may present various types of growth patterns and malocclusion, depending on the exact etiology of mouth breathing. Furthermore, breathing through the oral cavity can negatively affect oral health, increasing the risk of caries and periodontal diseases. This review aims to provide a summary of recent publications with regard to the impact of mouth breathing on dentofacial development, describe their consistencies and differences, and briefly discuss potential reasons behind inconsistent findings.

## Introduction

Mouth breathing is one of the most common deleterious oral habits in children and a symptom of sleep disordered breathing (SDB). Its prevalence ranges from 11 to 56% in children (1–4). As with other poor oral habits like abnormal biting habits, tongue habits, chewing habits, and sleeping habits (5), mouth breathing can disappear automatically with age. Otherwise, it may have a negative impact on children's dental and maxillofacial development (6).

Defined as over 25%−30% of the air passing through the mouth instead of the nose (7, 8), mouth breathing often occurs due to upper airway obstruction which reduces the nasal airflow and forces the air to enter completely or partially through oral cavity. According to the functional matrix theory established by Moss and Salentijn (9) in 1969, normal respiratory function of the nose is essential for the balanced growth of craniofacial structures. When upper airway obstruction is not removed promptly, or when mouth breathing is still habitually present after the removal of obstruction, mouth breathing will have negative effects on not only the normal development and function of the dentofacial complex but also the general health of growing children. Dentists, especially orthodontists who provide early treatment, play an important role during the growth period. The awareness of effective orthodontic prevention is therefore vital for dental professionals (10). Early screening and intervention of mouth breathing are beneficial for the normal development of dentofacial structure and function and can help prevent relevant harms to children's general health.

This review provides a succinct summary of recent publications regarding the impact of mouth breathing on dentofacial development, describes their consistencies and differences, in order to help dental practitioners and clinicians in other related specialties perform evidence-informed decision-making, and to improve the awareness of currently available evidence and key research findings among investigators and postgraduate students working in relevant fields.

## The etiology of mouth breathing

Mouth breathing can result from obstruction at any site of upper airway. Upper airway can be divided into four sections: nasal cavity, nasopharynx, oropharynx, and laryngopharynx (11). Different from the trachea and bronchi in lower airway, upper airway is not supported by hard tissues (12). Therefore, upper airway is directly influenced by the size, shape, and position of surrounding tissues (such as nasal mucosa, adenoids, and tonsils), and pathological changes in these tissues can interfere with the passage of airflow (13–15).

Nasal obstruction can be attributed to nasal inflammation in children, including allergic rhinitis, chronic rhinitis, and sinusitis. In recent years, environmental degradation and air pollution have led to an increased prevalence of nasal allergic diseases, and therefore allergic rhinitis related nasal obstruction has become more common. In addition, morphological deformities of the nose which affect nasal ventilation and reduce nasal airflow, such as the deviated nasal septum, turbinate hypertrophy, nasal polyps, and nasal trauma, can also lead to mouth breathing (16–18).

Adenotonsillar hypertrophy is the most common cause of mouth breathing in children (16, 19). The adenoid is lymphatic tissue of the posterior nasopharynx, while the palatine tonsils are in the fossa between the palatoglossal arch and pharyngopalatine arch. Both adenoids and palatine tonsils belong to the Waldeyer's ring (20). Adenoids are actively growing during the ages of 2–6 years and begin to decrease in size after 10 years old (21), while tonsils are generally most actively developing at 2–5 years old. Under normal physiological conditions, they gradually atrophy and disappear at the ages of 14–15 in the majority of people. However, pathological hypertrophic adenoids and tonsils are unable to atrophy normally. They will reduce pharyngeal cross-sectional area and block nasal breathing. Children need to breathe through the mouth completely or partially to access enough oxygen.

## The impact of mouth breathing on malocclusion

The function and morphology of the orofacial system are unified. The habitual position of muscles inside and outside the mouth will affect dental development (22). Malocclusion appears more frequently in mouth-breathing children than in nasal-breathing children (23). Children with normal breathing patterns keep their lips closed to form a sealed oral space. The tongue is positioned in contact with the palate and lingual side of maxillary dentition. A balanced muscle strength from the internal tongue and external lips and cheek is crucial for the development of a normal upper dental arch.

Mouth breathing results in muscle imbalance, which may lead to oral and craniofacial alterations. Mouth-breathing children have a significant decrease in tongue pressure (24, 25). Children with mouth breathing resulting from upper airway obstruction tend to have a downward position of the lingual muscles, which disturbs the balance, contributing to the compression of the upper dentition and constricted maxillary dental arch (26, 27), as well as the crossbite of posterior teeth (23). The mandible has a posterior rotation and the posterior teeth have an excessive eruption. Thus, a risk of an open bite increases (28–30). A cross-sectional study of 1,616 children aged 3–6 years found that mouth breathing was related to anterior open bite, posterior crossbite, and increased overjet (31). Another study of 86 children also found that large adenoids were significantly associated with anterior open bite (32).

**Figure 1:**
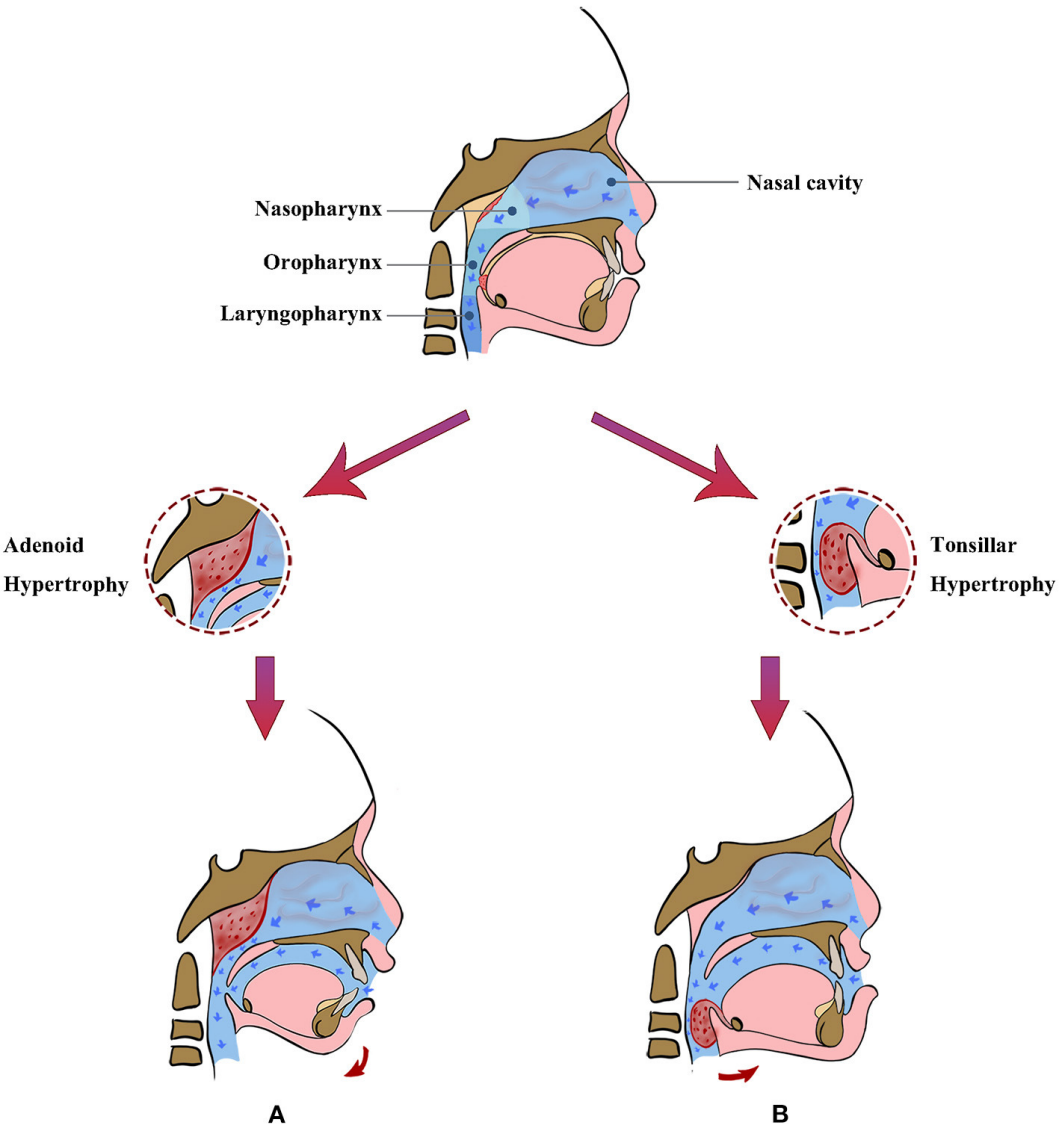
Mouth breathing resulted from adenoid hypertrophy or tonsillar hypertrophy may have different impact on dentofacial development in children. **(A)** Adenoid hypertrophy may lead to Class II malocclusion with an increased overjet and clockwise rotated mandible. **(B)** Tonsillar hypertrophy may result in mandibular protrusion, Class III malocclusion, and a tendency of anterior teeth crossbite.

The clinical features of malocclusion can vary with etiological factors (i.e., adenoid hypertrophy or tonsillar hypertrophy) of mouth breathing (Figure 1). Posterior nasopharyngeal obstruction due to pathological adenoid hypertrophy can lead to clockwise rotation of the mandible during mouth breathing, often presenting Class II malocclusion and large overjet (33, 34). Pathological hypertrophy of tonsils can obstruct the lower section of upper airway, making a child tend to move his/her mandible forward to increase the width of oropharyngeal airway, which often results in anterior crossbite (6, 35).

However, several studies on mouth breathing with adenoids and/or tonsils hypertrophy have also shown different conclusions. A previous study found that tonsillar hypertrophy in different degrees was associated with an increased risk of large overjet and Class II malocclusion, whereas adenoid hypertrophy was not significantly associated with malocclusion in children around 6 years of age (36). A possible explanation is that adenoid hypertrophy has a delayed effect on malocclusion, which did not occur at a younger age. A case–control study found that allergic rhinitis related nasal obstruction was a significant risk factor for the development of malocclusions (37). However, some systematic reviews explored the association between rhinitis-related mouth breathing and malocclusion in children, and concluded that the included studies had a high risk of bias and did not support a significant association (38, 39). Thus, even though the previous literature and studies have described the important influence of mouth breathing on malocclusion in children, related publications continue unabatedly to elaborate opposite views. In the future, mouth breathing should be managed based on etiologic considerations and comprehensive analysis of the malocclusion pattern, which may help to clarify the mechanisms of malocclusion more clearly.

## The impact of mouth breathing on maxillofacial development

In 1981, Harvold et al. (40) carried out a classical oral respiration experiment on rhesus monkeys. He built mouth-breathing models in rhesus monkeys by obstructing the nasal passages with silicon nose plugs. The monkey with nose plugs had to breathe through the mouth. After comparing the facial appearance and occlusion of experimental and control animals, he found that rhesus monkeys with nasal obstruction maintained a lower position of the mandible, a steeper mandibular plane, and an increase in facial height. The maxillofacial morphological changes occur because mouth breathing results in adaptive changes in the lips, tongue, and mandible, which further impact the skeletal alterations by neuromuscular responses. The traditional view is that children with mouth breathing often present with a skeletal Class II facial profile characterized as maxillary protrusion and mandibular retrusion (41, 42), along with the clockwise rotation of the mandible (28, 43), an increase in lower anterior height (44, 45), protruding upper lip (46), incompetent lip seal (47), nasal flaring and high palatal vault (48, 49). A systematic review of 19 studies compared cephalometric data in children and adolescents with oral and nasal breathing. Although the quality of the included studies was not high, there was evidence that retrognathic maxilla and mandible present more in mouth breathers, and they tend to have an increased angle of mandibular plane, as well as a downward and backward rotation mandible (50). Moreover, a high palatal vault is one of the most common characteristics in mouth-breathing patients, it has been shown that the palatal height in the molar region was 11% higher in mouth-breathing children than in those who breathe through the nose (27).

Similar to the malocclusion, skeletal patterns present dissimilarly with different etiological factors. The obstruction resulting from tonsillar hypertrophy often exhibits a Class III skeletal pattern, which is distinguished by maxillary sagittal dysplasia and mandibular protrusion (51–53). Iwasaki et al. (52) divided 64 children into two groups by ANB to explore the maxillofacial features affected by upper airway obstructive factors. This study indicated that hypertrophic tonsils with the anterior posture of tongue might induce mandibular protrusion. The lateral cephalometric radiographs of 226 children were collected in another study (53), which grouped the children by different categories of obstructive factors: adenoid, tonsils, and both of the tissues above. It found that children with isolated palatine tonsillar hypertrophy had a more horizontal mandible compared to children obstructed only by adenoids, and the position of the mandible was more forward, which was consistent with the occurrence of malocclusion with the same cause. Thus, mouth breathing tends to show different skeletal profiles (52), and a clear etiology may help to distinguish the maxillofacial growth direction.

However, some publications have stated different conclusions. It has also been suggested that mouth breathing has no impact on sagittal parameters but is significantly associated with lower anterior facial height in the vertical direction. Furthermore, the removal of hypertrophic adenoids improves vertical skeletal growth (54). A systematic review concluded that a significant correlation between children with OSA and maxillofacial abnormalities could not be established (55). According to the conclusion of another systematic review, it could not be identified yet that there was an association between mouth breathing and craniofacial and occlusal development (56). The current studies suffer from several methodological problems, such as the limits of sample size, geography, and ethnic selection, which may be the reason that no consensus can be reached temporarily. Future studies could consider mouth breathing as a single influential factor and make research protocols more rigorous to explore the relationship between breathing patterns and maxillofacial development. Also, there is an unequivocal necessity for evidence-based approaches.

## The impact of mouth breathing on dentofacial health

When air flows through the mouth, saliva evaporates and causes a decrease in the humidity in oral cavity. As an important agent for immune defense, antibacterial action, lubrication, and dissolution of inorganic substances, saliva contributes to the stability of PH in the oral environment and the prevention of dental caries (57). Many studies have indicated that children with chronic mouth breathing are at a higher risk of caries (58–61). Research has found significantly higher levels of streptococcus mutans and plaque in mouth-breathing adolescents (62). Mouth breathing can result in the alterations of saliva-mediated defense and the reduced self-cleaning effect of saliva, which leads to accelerated accumulation of plaque. In addition, the decrease of epithelial cells that can defend against plaque (60) and the dehydration of the gingival surface result of the airflow can also contribute to the development of gingivitis and other periodontal diseases (59).

In an intermittent nasal congestion rat model, there was a significant reduction in the thickness of the hyperplasia layer as well as the hypertrophic layer of condylar joint, which suggested that the development of the temporomandibular joint was also influenced by oral breathing habits (63). A previous randomized controlled trial (64) established another bilateral rat model of intermittent nasal congestion, and it found that mouth breathing would lead to defects in condylar development during adolescence, with the mechanism that chondrogenic differentiation of condylar mesenchymal stem cells was inhibited. Additionally, mouth breathing has been found to correlate with upper respiratory tract infections (65), halitosis (66, 67) and bruxism (68–70) in children, while bruxism may aggravate temporomandibular disorders (71) and excessive attrition of tooth enamel, which may lead to the malocclusion. However, the mechanisms of the effects of mouth breathing on caries, the health of periodontal tissues, and the temporomandibular joint are not clear yet. There are many confounding factors, such as the interrelationship between mouth breathing and malocclusion, which can also affect the health of the temporomandibular joint as well as periodontal tissues. In addition, abnormalities in the dentofacial areas can also be causally related to each other, which is a direction that can be explored further.

## Discussion

Mouth breathing is a common deleterious oral habit among children. During the growth period, mouth breathing may negatively affect dentofacial development if not corrected in time. Consequences resulting from mouth breathing habits include malocclusion, the deterioration of oral hygiene, increased prevalence of caries, periodontal diseases, and abnormal maxillofacial growth. Therefore, the early diagnosis and intervention of mouth breathing, based on a thorough analysis of its exact etiology, is of paramount importance (72).

Adenotonsillar hypertrophy is the most common cause of mouth breathing in children. Adenoids and palatine tonsils are located in different sites of upper airway. During the growth period, the obstruction occurring in different locations and times may result in corresponding facial patterns. Thus, the dentofacial development of children with both tonsillar hypertrophy and adenoid hypertrophy presents great complexity. Adenotonsillectomy may promote the normalization of breathing patterns and inhibit or even reverse the development of dentofacial deformity during the growth period (73, 74). It has been found that the need for secondary surgery after adenoidectomy or tonsillectomy is common (75–77), which may be associated with compensatory hypertrophy of residual lymphoid tissue. Thus, removing both lymphoid tissues at the same time is usually preferable.

This review aims to provide a summary of recent publications with regard to the impact of mouth breathing on dentofacial development, describe their consistencies and differences, and briefly discuss potential reasons behind inconsistent findings. Actually, for more than 100 years since mouth breathing was proposed, its influence on the malocclusion and morphological and functional development of the maxillofacial region has been controversial. What remains unknown is the precise contribution of genetic and environmental factors. In recent years, new relevant studies elaborate their findings and offer different opinions. What counts is that there is no high-quality evidence elucidating the effects of mouth breathing on dentofacial development and health, which is also due to the lack of well-designed clinical studies. The mechanism of mouth-breathing impact on the development of the dental and craniofacial region is still unclear.

Many studies have relied on questionnaires, inspection, simple screening tests (such as water holding test and mirror test), and the diagnosis of nasal endoscopy used by otolaryngologists to identify mouth-breathing patients. To our knowledge, there are no strict criteria for the diagnosis of mouth breathing, and the subjective assessment of mouth breathing may be one of the reasons why the findings are difficult to achieve agreement. The lack of diagnostic criteria makes the research on the association between mouth breathing and dentofacial development limited as well. In the future, more comprehensive studies should also be devoted to the establishment of a unified guideline of mouth breathing diagnostic criteria, as well as improvement of research methods, and further exploration of the effects and mechanisms of mouth breathing. The aim is to advance the knowledge of clinical dentists and pediatricians about mouth breathing and its influences, furthermore, to provide them with a more evidence-based diagnostic paradigm. Early screening of children's potential mouth breathing habits can help to interrupt it before their growth spurt, thus avoiding possible adverse impacts.

## Author contributions

LL and TZ: manuscript drafting. LL: visualization. DQ, FH, and HH: critical revision of the manuscript. All authors: approval of the final version.

## Funding

This work was supported by the Wuhan Knowledge Innovation Project (No. 2022020801020502), the CSA Orthodontic Clinical Research Project for Central and West China (No. CSA-MWO2021-01), the Fundamental Research Funds for the Central Universities (No. 2042021kf0182, Wuhan University), and the Wuhan University School & Hospital of Stomatology Clinical Research Project (No. LYZX202101).

## Conflict of interest

The authors declare that the research was conducted in the absence of any commercial or financial relationships that could be construed as a potential conflict of interest.

## Publisher's note

All claims expressed in this article are solely those of the authors and do not necessarily represent those of their affiliated organizations, or those of the publisher, the editors and the reviewers. Any product that may be evaluated in this article, or claim that may be made by its manufacturer, is not guaranteed or endorsed by the publisher.
